# Patient-reported outcomes of the different prescribing patterns in heart failure with reduced and mildly reduced ejection fraction

**DOI:** 10.1108/IJHCQA-08-2025-0122

**Published:** 2026-04-16

**Authors:** Mohamed Metwally Mosly, Enas Elkady, Seif El Hadidi, Rasha El Sorady, Fakhr Al Ayoubi, Engy Emam, Rabab Kosba, Ahmed Abdel Aaty

**Affiliations:** Arab Academy for Science Technology and Maritime Transport, Alexandria, Egypt; Alexandria Main University Hospital, Alexandria, Egypt; School of Population Health, Royal College of Surgeons in Ireland, Dublin, Ireland; National Office of Clinical Audit, Dublin, Ireland; King Saud University, Riyadh, Saudi Arabia; Faculty of Medicine, Alexandria University, Alexandria, Egypt

**Keywords:** Heart failure, HFrEF, HFmrEF, Quality of life, Guideline-directed medical therapy, MLHFQ, ARNi, SGLT2 inhibitors, Patient-reported outcomes

## Abstract

**Purpose:**

To evaluate whether different real-world prescribing patterns of guideline-directed medical therapy (GDMT) in patients with heart failure with reduced ejection fraction (HFrEF) or mildly reduced ejection fraction (HFmrEF) are associated with differences in patient-reported quality of life (QoL), and specifically to compare conventional triple therapy, ARNi-based therapy, SGLT2i-based therapy, and full quadruple GDMT.

**Design/methodology/approach:**

This was a multicentre, cross-sectional descriptive study conducted in two tertiary care centres in Egypt and Saudi Arabia between December 2022 and March 2024. A total of 118 adult patients with LVEF <50% were enrolled at their first follow-up visit after hospital discharge. Participants were grouped according to prescribed HF regimen: conventional triple therapy, ARNi-based therapy, SGLT2i-based therapy, or quadruple GDMT. Quality of life was measured using the Minnesota Living with Heart Failure Questionnaire (MLHFQ) 7–14 days after discharge. Between-group differences were analysed using one-way ANOVA with Tukey HSD post hoc testing, and multivariable linear regression was used to identify predictors of MLHFQ score.

**Findings:**

Quality-of-life scores differed significantly across the treatment groups. Patients receiving quadruple GDMT had the best QoL, reflected by the lowest mean MLHFQ score (42.77 ± 19.05), whereas those receiving conventional triple therapy had the worst QoL (68.06 ± 19.77). ANOVA showed a statistically significant overall difference between regimens (*F*(3,114) = 8.135, *p* < 0.001). Post hoc analysis showed significantly better QoL with quadruple GDMT versus conventional triple therapy, and versus the SGLT2i-based triple regimen. In regression analysis, higher serum creatinine and blood urea nitrogen were independently associated with worse QoL, while higher haemoglobin was associated with better QoL. The study also found that patients receiving quadruple GDMT had shorter hospital stays compared with those receiving other regimens.

**Research limitations/implications:**

The cross-sectional design limits causal inference and temporal interpretation of GDMT effects on quality of life. Residual confounding is possible due to unmeasured factors such as disease severity, medication adherence, duration of therapy, and socioeconomic status. The relatively small sample size and limited geographic scope may affect generalisability. Clinically, the findings support systematic optimisation of GDMT and routine integration of patient-reported outcomes (e.g. MLHFQ) into care. They also highlight the importance of managing renal dysfunction and anaemia to improve QoL and justify further longitudinal and interventional research. Future multicentre, longitudinal studies are warranted to validate these findings and evaluate cost-effectiveness and long-term adherence.

**Practical implications:**

Clinicians should prioritise early optimisation of full GDMT, particularly incorporating ARNi and SGLT2 inhibitors, where tolerated, to enhance patient-reported quality of life. Routine use of validated tools such as the MLHFQ during follow-up can guide treatment adjustments. Multidisciplinary care – especially pharmacist-led medication reconciliation – may improve GDMT uptake and adherence. Regular monitoring and management of renal function and anaemia are essential to optimise outcomes. Shared decision-making should be emphasised to balance treatment complexity with patient preferences and improve adherence in real-world settings.

**Social implications:**

Improved optimisation of GDMT may enhance patients' functional status, independence, and ability to participate in daily, social, and occupational activities, thereby reducing caregiver burden and societal costs. Better quality of life and fewer hospitalisations can decrease healthcare resource utilisation and economic strain on health systems. Emphasising patient-reported outcomes supports more equitable, patient-centred care, particularly in diverse and resource-variable settings, helping to reduce disparities in heart failure management and long-term outcomes.

**Originality/value:**

The study provides novel real-world evidence from the Middle East and Africa on the association between contemporary GDMT combinations and early patient-reported QoL after discharge. Its main value lies in moving beyond traditional clinical endpoints such as mortality and hospitalisation to examine the lived experience of patients receiving different HF regimens. The authors position it as the first multicentre post-discharge study from this region to directly compare QoL across conventional triple therapy, ARNi-based therapy, SGLT2i-based therapy, and full quadruple GDMT.

## Introduction

Heart failure with reduced and mildly-reduced ejection fraction (HFrEF and HFmrEF) remains a critical global health concern with rising incidence and considerable clinical and economic burdens. (Pratley *et al*., 2024; El-Hadidi *et al*., 2026) According to the American Heart Association, approximately 6.2 million adults in the United States alone are living with HF, with projections indicating a substantial increase in prevalence in the coming decades. (Braunschweig *et al*., 2011) This necessitates ongoing research to refine therapeutic strategies that address validated metrics for patient-reported outcomes and lived experiences (PROMs and PREMs). (Stolfo *et al*., 2025; Abdin *et al*., 2025; Nicola *et al*., 2024)

Traditionally, HF management has relied on evidence-based polypharmacy targeting various aspects of the disease pathologies. (Stolfo *et al*., 2025; El Hadidi *et al*., 2020, 2021) These include angiotensin-converting enzyme inhibitors (ACEi), evidence-based beta-blockers, and mineralocorticoid receptor antagonists (MRAs), which together have been shown to reduce hospitalisations and mortality. (Yancy *et al*., 2017; El Hadidi *et al*., 2018) Recent advances have introduced novel appropriate polypharmacy and dosage forms, including angiotensin receptor–neprilysin inhibitors (ARNi) and sodium–glucose cotransporter-2 inhibitors (SGLT2i), which have substantially transformed the management of heart failure with reduced and mildly reduced ejection fraction (HFrEF and HFmrEF) and improved patient care pathways. (El-Hadidi *et al*., 2026; Monzo *et al*., 2025; El Hadidi and Rosano, 2020) Notably, the PARADIGM-HF trial demonstrated that sacubitril/valsartan (Entresto®) was superior to enalapril in reducing cardiovascular mortality and heart failure hospitalisations, representing a major advance in heart failure therapy. (McMurray *et al*., 2014)

Moreover, SGLT2 inhibitors, initially developed as antihyperglycemic agents, have demonstrated significant benefits in HF patients regardless of diabetes status. The DAPA-HF and EMPEROR-Reduced trials have consistently reported reductions in HF-related events and improvements in quality of life (QoL) with dapagliflozin and empagliflozin, respectively. (Docherty *et al*., 2020; Anker *et al*., 2021) These findings have expanded the therapeutic options available, providing new possibilities for smartly optimising HF management. The compelling evidence from these trials emphasises the clinical advantages of combination therapy involving ARNi and SGLT2 inhibitors to enhance patient outcomes synergistically. (Tadic *et al*., 2024)

Despite strong clinical trial evidence supporting contemporary guideline-directed medical therapy (GDMT), real-world data comparing the impact of the different and commonly prescribed therapeutic regimens on patient-reported quality of life and lived experiences remain limited, particularly during the early post-discharge period. (Hadidi *et al*., 2022; Nicola *et al*., 2024)

Therefore, this study was undertaken to examine whether the commonly prescribed combinations are associated with meaningful differences in patients' lived experiences outside controlled trial settings. By prioritising patient-reported outcomes rather than clinical endpoints alone, this study provides novel real-world insight into how contemporary prescribing practices are reflected in the lived experience and early recovery of patients with HFrEF and HFmrEF.

## Method

### Study design

A cross-sectional descriptive study was conducted at two Middle Eastern tertiary care centres in Egypt and Saudi Arabia between December 2022 and March 2024 upon their first follow-up visits after discharge. This study is reported in accordance with the STROBE (Strengthening the Reporting of Observational Studies in Epidemiology) recommendations for cross-sectional studies. (von Elm *et al*., 2007)

The study aimed to compare the MLHFQ total score among patients receiving the triple therapy regimens (β-blocker + MRA and ACEi or ARBs), (β-blocker + MRA and ARNi), (β-blocker + MRA and SGLT2 inhibitors), and the recommended quadruple combination of GDMT (β-blocker + MRA + ARNi and SGLT2 inhibitors)

As a real-world, cross-sectional observational study, the objective is not to test a controlled intervention but to compare naturally occurring prescribing patterns and their association with QoL in routine clinical practice. This design is methodologically appropriate for exploratory comparative effectiveness research and for capturing patient-reported outcomes outside trial settings.

### Eligibility criteria

All patients ≥18 years with heart failure and left ventricular ejection fraction (LVEF) < 50% (reduced or mildly reduced EF) receiving one of four therapeutic regimens were included.

Exclusion criteria included all the following conditions: HF with preserved ejection fraction, end-stage renal disease, end-stage liver disease, cancer, and prior heart transplantation.

### Study groups

1. *Conventional Triple Therapy regimen*: β-blocker + MRA + ACEi/ARB
2. *ARNi-Based Triple regimen*: β-blocker + MRA + ARNi
3. *SGLT2i-Based Triple regimen*: β-blocker + MRA + SGLT2i
4. *Quadruple regimen (GDMT)*: β-blocker + MRA + ARNi + SGLT2i

### Data collection

Demographic and clinical variables were extracted from electronic medical records by the research team using a standardised data-collection sheet. All laboratory results corresponded to the index hospital admission, whereas MLHFQ was administered at the first outpatient follow-up (7–14 days post-discharge) During the same visit, a trained investigator conducted a face-to-face interview to ensure the complete responses of the questionnaire.

### Outcome measures

The QoL was assessed using the Minnesota Living with Heart Failure Questionnaire (MLHFQ) administered at the first cardiology outpatient follow-up, 7–14 days after discharge. (Behlouli *et al*., 2009) A face-to-face interview was conducted for questionnaire collecting, a validated tool specifically designed to measure the impact of HF on patients' lives. (Bilbao *et al*., 2016; Naveiro-Rilo *et al*., 2010)

Scores are judged as follows: Lower MLHFQ scores indicate better QoL. For descriptive categorisation, we classified <24 as good, 24–45 as moderate, and ≥45 as poor QoL in line with prior validation work.

## Statistical analysis

All statistical analyses were performed using IBM SPSS Statistics for Windows, Version 27.0. Armonk, NY: IBM Corp., USA.

*Descriptive statistics* were used to summarise the baseline characteristics of the study population, including means and standard deviations for continuous variables and frequencies and percentages for categorical variables.

### Inferential statistics

A one-way between-groups analysis of variance (ANOVA) was conducted to determine if there were statistically significant differences in MLHFQ total score among the four therapeutic regimen groups. The independent variable was the therapeutic regimen, and the dependent variable was the MLHFQ score.

Following a significant ANOVA result, Tukey's Honestly Significant Difference (HSD) test was performed for post hoc comparisons to identify specific group differences. A multiple linear regression analysis was conducted to assess the ability of the independent variables (platelet count, ejection fraction (EF%), haemoglobin, Serum creatinine, sex (male/female), white blood cells (WBCs), and blood urea nitrogen (BUN) to predict the value of the MLHFQ score. Statistical significance was judged at a 5% level. We screened continuous variables for implausible values and unit mismatches. Creatinine values > 15 mg/dL or <0.3 mg/dL were considered implausible and handled via predefined rules (verification against source chart; if unresolved, casewise exclusion for analyses involving creatinine). For normally distributed continuous outcomes, we report the mean (SD) and 95% CI of the mean. Group differences were assessed by one-way ANOVA with Tukey HSD for pairwise comparisons. Model assumptions were evaluated via residual diagnostics.

### Ethical considerations

Written informed consent was obtained from all participants before their voluntary participation in the study. The study was approved by the two local research ethics committees of the two hospitals in the two countries, in line with the Declaration of Helsinki and adhered to good clinical practice standards.

### Data management

Data were fully anonymised to ensure patient privacy and confidentiality. All data were stored in a secure, dual password-encrypted database accessible only to the research team. Regular data audits were conducted to maintain data integrity and accuracy in the two countries.

## Results

### Descriptive analysis

From the two countries, the study enrolled a total of 118 voluntary HFrEF/HFmrEF patients who completed the questionnaire in full. The demographic and clinical characteristics are summarised in Table 1. The mean ejection fraction (EF) was 29.8% (SD = 8.1), and the mean serum creatinine was 3.941 mg/dL (SD = 1.5). The mean blood urea nitrogen level was 49.6 mg/dL (SD = 39.2), and the mean haemoglobin was 12 g/dL (SD = 2.3). White blood cell (WBC) count averaged 7.6 × 10ˆ3/µL (SD = 2.8), and platelet count averaged 236.7 × 10ˆ3/µL (SD = 75.2) (see Table 2).

**Table 1:** Baseline characteristics of the study population

Variable	Mean ± SD	Minimum	Maximum
Ejection Fraction (%)	29.8 ± 8.1	15	45
Serum Creatinine (mg/dL)	3.9 ± 1.5	0.3	6.7
Urea (mg/dL)	49.6 ± 39.2	5	236
Haemoglobin (g/dL)	12.0 ± 2.3	8	18.9
White Blood Cells ( × 10ˆ3/µL)	7.6 ± 2.8	3	18
Platelets ( × 10ˆ3/µL)	236.7 ± 75.2	41	453
MLHFQ total score	56.5 ± 22.1	7	98

**Note(s):** MLHFQ: minnesota living with heart failure questionnaire

**Table 2:** MLHFQ scores of the four commonly prescribed therapeutic regimens

Regimen	Mean MLHFQ total score ±SD	Minimum – Maximum range
Conventional Triple regimen	68.1 ± 19.8	26–98
ARNi-based triple regimen	57.3 ± 19.9	7–87
SGLT2i-based triple regimen	56.9 ± 22.2	21–92
Quadruple regimen (GDMT)	42.8 ± 19.0	9–92

**Note(s):** ARNi: Angiotensin Receptor-Neprilysin Inhibitor; GDMT: guideline-directed medical therapy; SD: standard deviation; SGLT-2i: sodium-glucose cotransporter 2 inhibitors

**Figure 1:**
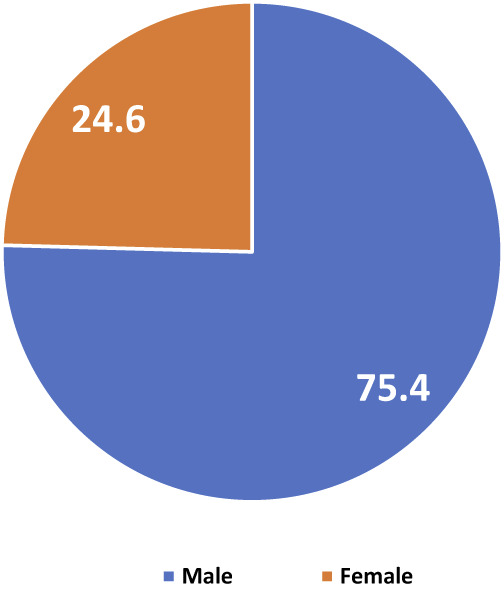
Respondents' sex distribution %

The average score on the Minnesota Living with Heart Failure Quality of Life Questionnaire was 56.50 (SD = 22.113). Sex distribution showed 89 males (75.4%) and 29 females (24.6%), as in Figure 1.

**Figure 2:**
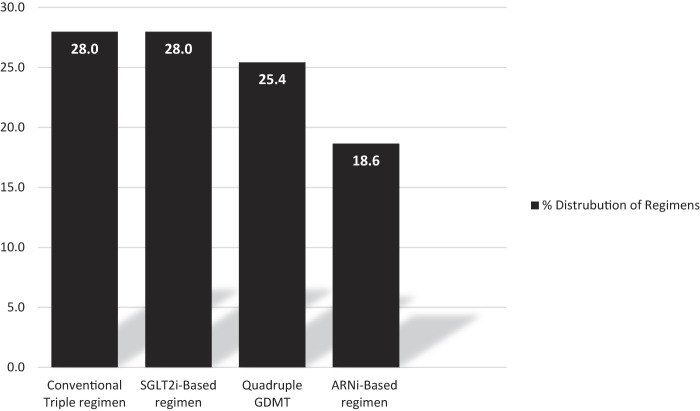
The prescription rates of the four distinct therapeutic regimens

Figure 2 shows the prescription rates of the four distinct therapeutic regimens that were commonly prescribed among the study population. Group 1: 33 (28%) patients received conventional triple therapy (β-blockers, MRA, and ACEi or ARBs). The second group comprised 22 patients (18.6%) who received a triple regimen containing a β-blocker, MRA, and ARNi (Sacubitril/Valsartan) as an ARNi-based therapy. Another group was 33 patients (28%) who were administered a β-blocker, MRA, and SGLT2i as an SGLT2i-based triple regimen. The fourth group was 30 patients (25.4%) treated with the full four pillars (β-blockers, MRA, ARNi and SGLT2i) as the quadruple GDMT.

### Inferential statistics

The conventional triple therapy group had the highest mean MLHFQ score (68.06 ± 19.77), whereas the quadruple GDMT group had the lowest mean score (42.77 ± 19.05), indicating better QoL.

Applying ANOVA for comparison between the 4 regimens regarding MLHFQ score revealed a statistically significant difference between the four groups (*p* < 0.001), as in Table 3.

**Table 3:** ANOVA for MLHFQ scores between different regimens

	Sum of squares	df	Mean square	*F*	Sig.
Between Groups	10087.967	3	3362.656	8.135	<0.001
Within Groups	47121.533	114	413.347		
Total	57209.500	117			

Post-hoc pairwise comparison in Table 4 declared the statistically significant difference between group one, the conventional triple therapy, and group four patients who received the four pillars of GDMT (*p* < 0.001), also the statistically significant difference between patients' group 3 received SGLT2i with (Β-blocker and MRA) and group 4 received the 4 pillars (*p* = 0.036).

**Table 4:** Post hoc pairwise multiple comparisons by Tukey HSD test between different regimens

(I) Regimen	(J) Regimen	Mean difference (I—J)	Std. error	Sig.	95% confidence interval	
					Lower bound	Upper bound
Conventional triple regimen	ARNi	10.742	5.596	0.226	−3.85	25.33
	SGLT2i	11.182	5.005	0.120	−1.87	24.23
	Quadruple	25.294^*^	5.129	*<0.001*	11.92	38.67
ARNi-based triple regimen	Conventional	−10.742	5.596	0.226	−25.33	3.85
	SGLT2i	0.439	5.596	1.000	−14.15	15.03
	Quadruple	14.552	5.707	0.058	−0.33	29.43
SGLT2i-based triple regimen	Conventional	−11.182	5.005	0.120	−24.23	1.87
	ARNi	−0.439	5.596	1.000	−15.03	14.15
	Quadruple	14.112^*^	5.129	*0.034*	0.74	27.48

**Note(s):** ARNi: Angiotensin Receptor-Neprilysin Inhibitor; GDMT: guideline-directed medical therapy; SGLT-2i: sodium-glucose cotransporter 2 inhibitors

^*^Statistical significance difference (*P*-value < 0.05)

Tables 5 and 6 show that the length of hospital stay differed significantly among regimens (one-way ANOVA, *F* = 5.361, *p* = 0.004). Tukey HSD post hoc analysis showed that patients receiving conventional triple therapy had significantly longer hospital stay than those receiving the SGLT2i-based regimen (mean difference 1.333 days, 95% CI 0.47 to 2.20, *p* = 0.001) and quadruple GDMT (mean difference 1.933 days, 95% CI 1.05 to 2.82, *p* < 0.001). In addition, patients receiving ARNi-based therapy had a longer stay than those receiving quadruple GDMT (mean difference 1.085 days, 95% CI 0.10 to 2.07, *p* = 0.025).

**Table 5:** ANOVA for length of hospital stay between the different regimens

	Sum of squares	Mean square	*F*	Sig.
Between Groups	23.434	7.811	5.361	0.004
Within Groups	52.469	1.457		
Total	75.902			

**Table 6:** Length of hospital stay

(I) Regimen	(J) Regimen	Mean difference (I−J)	Std. error	Sig.	95% CI lower	95% CI upper
Conventional triple therapy	ARNi-based regimen	0.848	0.372	0.108	−0.12	1.82
	SGLT2i-based regimen	*1.333*	0.332	*0.001*	0.47	2.20
	Quadruple GDMT	*1.933*	0.341	*<0.001*	1.05	2.82
ARNi-based regimen	Conventional triple therapy	−0.848	0.372	0.108	−1.82	0.12
	SGLT2i-based regimen	0.485	0.372	0.562	−0.48	1.45
	Quadruple GDMT	*1.085*	0.379	*0.025*	0.10	2.07
SGLT2i-based regimen	Conventional triple therapy	*−1.333*	0.332	*0.001*	−2.20	−0.47
	ARNi-based regimen	−0.485	0.372	0.562	−1.45	0.48
	Quadruple GDMT	0.600	0.341	0.297	−0.29	1.49

**Note(s):** ARNi: Angiotensin Receptor-Neprilysin Inhibitor; GDMT: guideline-directed medical therapy; SGLT-2i: sodium-glucose cotransporter 2 inhibitors

Table 7 showed the statistically significant difference between groups regarding the EF% (*p* = 0.047). Patients receiving quadruple GDMT had lower EF than those receiving conventional triple therapy (mean difference 5.27% points, 95% CI 0.10 to 10.43, *p* = 0.044) as seen in Table 8.

**Table 7:** ANOVA for EF% between different regimens

	Sum of squares	df	Mean square	*F*	Sig.
Between groups	503.731	3	167.910	2.725	0.047
Within groups	7023.973	114	61.614		
Total	7527.703	117			

**Table 8:** Post hoc pairwise comparison concerning the EF% between regimens (Tukey HSD) in the four groups

(I) Regimen	(J) Regimen	Mean difference (I—J)	Std. error	Sig.	95% confidence interval	
					Lower bound	Upper bound
Conventional	*ARNi regimen*	3.864	2.160	0.284	−1.77	9.50
	*SGLT2i regimen*	4.121	1.932	0.149	−0.92	9.16
	*Quadruple GDMT*	5.267^*^	1.980	0.044	0.10	10.43

**Note(s):** ARNi: Angiotensin Receptor-Neprilysin Inhibitor; GDMT: guideline-directed medical therapy; SGLT-2i: sodium-glucose cotransporter 2 inhibitors

### Regression analysis

A multiple linear regression model was used to examine independent predictors of MLHFQ score. The overall model was statistically significant, *F = 5.234, p < 0.001*, and explained *25.0%* of the variance in MLHFQ score (*R*^*2*^ *= 0.250; adjusted R*^*2*^ *= 0.202*), Table 9.

**Table 9:** Regression analysis parameters for the effect of independent variables on the MLHFQ scoring

R	*R* ^2^	Adjusted *R*^2^	Std. Error of estimate	*F*	df1	df2	*p*
0.500	0.250	0.202	19.752	5.234	7	110	<0.001

Higher serum creatinine (*B = 0.295, p = 0.010*) and blood urea nitrogen (*B = 0.157, p = 0.003*) were independently associated with higher (worse) MLHFQ scores, whereas higher haemoglobin (*B = −2.314, p = 0.011*) was independently associated with lower (better) MLHFQ scores, Table 10.

**Table 10:** Predictors of MLHFQ scoring

Variable	Unstandardized coefficients		Standardized coefficients	*t*	Sig.
	B	Std. Error	Beta		
(Constant)	79.367	14.487		5.479	*<0.001*
Sex	−4.57	4.511	−0.089	−1.013	0.313
Ejection fraction	−0.008	0.234	−0.003	−0.032	0.974
Serum creatinine	0.295	0.113	0.223	2.619	*0.01*
Blood Urea Nitrogen (BUN)	0.157	0.051	0.279	3.059	*0.003*
Haemoglobin (HgB)	−2.314	0.899	−0.24	−2.573	*0.011*
White blood cells	−1.317	0.714	−0.165	−1.844	0.068
Platelets	0.031	0.026	0.105	1.173	0.243

For clinical interpretability, the simplified predictive relationship can be expressed as:


Predicted MLHFQ score=79.367+0.295×(Serum Creatinine)+0.157×(BUN)−2.314×(Haemoglobin)

## Discussion

This cross-sectional study examined the association between guideline-directed medical therapy regimens and health-related quality of life in patients with HFrEF/HFmrEF, reflecting real-world outpatient practice and complementing existing evidence on patient-reported outcomes in heart failure. The presented findings corroborate the supporting evidence from the latest American and European clinical practice guidelines and advocate early initiation of all four GDMT pillars at the earliest convenience. (McDonagh *et al*., 2021; El-Hadidi *et al*., 2026) Such a finding is also in line with prior landmark studies, such as the DAPA-HF and EMPEROR-Reduced trials, which highlighted the synergistic benefits of SGLT2 inhibitors alongside conventional HF treatments. (Docherty *et al*., 2020; Anker *et al*., 2021; El Hadidi *et al*., 2021) These benefits might stem from the complementary mechanisms of action, combining natriuretic and diuretic effects with inhibition of the renin-angiotensin system.

The high MLHFQ scores among patients on conventional triple therapy, despite previously established mortality benefits, may be attributed to residual symptomatology. Despite its comprehensive scope, the conventional triple therapy group exhibited the highest MLHFQ scores, indicating poorer QoL outcomes. Previous literature, including guidelines by Yancy *et al*., supports the effectiveness of beta-blockers, MRAs, and ACE inhibitors or ARBs in reducing HF hospitalisations. (Severino *et al*., 2024; El Hadidi *et al*., 2018) However, the study underscores that such benefits may not translate into improvements in QoL, potentially due to clinical inertia and fear of adverse effects or increased pill burden (Moradi *et al*., 2020). This finding is consistent with the hypothesis that the newer disease-modifying agents offer not only beneficial clinical stability but also enhanced patient-reported outcomes and experiences. (Shchendrygina and Saldarriaga, 2024)

Patients treated solely with ARNi or SGLT2 inhibitors demonstrated moderate improvements in QoL, consistent with evidence from pivotal trials such as PARADIGM-HF and DAPA-HF. (Kosiborod *et al*., 2020; El-Hadidi *et al*., 2026) While both therapies individually reduce cardiovascular mortality and hospitalisations, this study highlights that their effects on QoL, though significant, do not match the enhancements achieved through their combination. (Trueman *et al*., 2017) This finding reinforces the need for tailored therapeutic strategies tailored to patient-specific needs and experiences. (Stolfo *et al*., 2025; Nicola *et al*., 2024)

The significant reduction in hospital length of stay for patients on the ARNi and SGLT2 inhibitor combination further supports the clinical superiority of this regimen. These findings align with the international guidelines emphasising early initiation of combination therapy to optimise outcomes. Shorter hospital stays will also be translated into reduced healthcare costs and fewer disruptions to patients' daily lives, adding an economic dimension to the therapy's clinical benefits. (McEwan *et al*., 2021)

Regression analysis identified higher serum creatinine and blood urea nitrogen as predictors of worse QoL, whereas higher haemoglobin predicted better QoL. These markers highlight the importance of the appropriate management and correction of renal function, anaemia and any potentially inappropriate polypharmacy in HF patients. (El Hadidi *et al*., 2022b) The role of haemoglobin, in particular, suggests that addressing underlying anaemia could further enhance QoL outcomes. These findings corroborate prior research advocating the regular integration of renal and haematological evaluations into HF management protocols. (Oswald *et al*., 2024; Zhou *et al*., 2024)

When compared to global findings, the results resonate with trends observed in multicentre studies from Western European and Asian cohorts. (Lam *et al*., 2016) However, the context-specific nature of this study, involving Middle Eastern patients from Egypt as a low-income country from Africa and Saudi Arabia as a high-income country from Asia, provides unique insights into regional variations in patient outcomes and responses to therapy. (Hassanin *et al*., 2020; El Hadidi *et al*., 2020) These insights underscore the importance of conducting culturally and geographically diverse studies to refine global HF management strategies. (Dokainish *et al*., 2016)

The findings advocate for the early implementation of combination therapies, particularly ARNi and SGLT2 inhibitors, in clinical practice. (Cavallari *et al*., 2023; Tadic *et al*., 2024) Therefore, this study opens the room for the early inclusion of clinical pharmacy in the HF management team to achieve an appropriate medication therapy management during hospitalisation and the full prescription of the updated guideline-directed medical therapies upon discharge. (El Hadidi *et al*., 2020, 2022a; Alsetohy *et al*., 2025)

Patient-centred care models should integrate QoL systematically as a core parameter alongside clinical endpoints. Future research should prioritise multicentre, longitudinal studies to validate these results and explore cost-effectiveness over 5 and 10 years of therapy. (Halatchev *et al*., 2020; El Hadidi *et al*., 2020) Additionally, real-world data on long-term patients' adherence and patient-reported outcomes and experiences will be crucial to determining the sustainability of these benefits.

### Implications for clinical practice, research, and patient-centred care

The present findings have several important implications for clinical practice, research, and patient-centred care, while remaining consistent with the cross-sectional design of the study. The observed association between more comprehensive GDMT regimens and lower (better) MLHFQ scores suggests that, in real-world outpatient settings, patients receiving a greater number of guideline-recommended therapies tend to report better health-related quality of life. Although causality cannot be inferred, these results underscore the potential value of systematic GDMT optimization and careful medication reconciliation during follow-up visits, particularly for patients with persistently poor quality-of-life scores.

From a clinical implementation perspective, the findings support the integration of routine quality-of-life assessment, such as the MLHFQ, into heart failure care pathways to help identify patients who may benefit from closer evaluation of GDMT completeness or tolerability. In practice, clinicians may use patient-reported outcomes alongside clinical and laboratory parameters to guide individualised treatment discussions, especially when balancing the benefits of GDMT intensification against comorbidities, renal function, and patient preferences.

In terms of patient-centred care, the association between GDMT intensity and quality of life highlights the importance of shared decision-making. Discussing expected symptom burden and quality-of-life outcomes with patients may enhance engagement, adherence, and satisfaction with therapy, particularly in populations where polypharmacy or adverse effects are concerns.

From a research perspective, these findings generate hypotheses for future longitudinal and interventional studies. Prospective studies are needed to determine whether stepwise GDMT optimisation leads to sustained improvements in quality of life over time and to clarify the temporal relationship between laboratory markers, treatment intensity, and patient-reported outcomes. Randomized or pragmatic trials incorporating quality-of-life endpoints would further strengthen the evidence base and inform guideline implementation strategies.

### Strengths and limitations

This study offers important real-world insights into the association between contemporary GDMT regimens and patient-reported quality of life. Also, the multicentre design across two largely populated countries enhanced the outcome of the validated MLHFQ tool, the rigorous statistical analysis, and therefore, the generalisability and robustness of findings across the neighbouring countries. Additionally, by integrating biochemical and clinical predictors into regression modelling, the study provides a nuanced understanding of factors influencing QoL beyond pharmacological treatment.

However, some limitations must be acknowledged. First, the cross-sectional design precludes causal inference or determination of temporal relationships between GDMT regimens and quality-of-life outcomes; the observed associations reflect a single time point and may be influenced by reverse causality. Second, although we adjusted for selected clinical and laboratory variables, residual confounding remains possible. Important factors such as overall disease severity (including symptom burden and functional capacity), medication adherence, duration of therapy, and socioeconomic determinants of health (e.g., income, education, access to care, and social support) were not fully captured. They may independently influence patient-reported quality of life.

## Conclusion

In this cross-sectional analysis, the full GDMT regimen is associated with better patient-reported quality of life among patients with HFrEF/HFmrEF. While these findings suggest potential quality-of-life benefits for patients receiving multiple guideline-recommended therapies, causal inferences cannot be drawn, and treatment decisions should be individualised based on clinical characteristics, comorbidities, and patient preferences.

## Data Availability

The data underlying this article will be shared on reasonable request to the corresponding author.

## References

[ref001] Abdin, A., Bauersachs, J., Abdelhamid, M., Aktaa, S., Al Ghorani, H., Bayes-Genis, A., Biegus, J., Bohm, M., Butler, J., Girerd, N., Metra, M., Mullens, W., Skouri, H., Vaduganathan, M., El Hadidi, S., Rosano, G.M.C. and Savarese, G. (2025), “Pharmacologic pitfalls in heart failure: a guide to drugs that may cause or exacerbate heart failure. A European journal of heart failure expert consensus document”, European Journal of Heart Failure, Vol. 27 No. 12, pp. 2671-2690, doi: 10.1002/ejhf.70087. 41382384 PMC12803569

[ref002] Alsetohy, W.M., El-Fass, K.A., El Hadidi, S., Zaitoun, M.F., Badary, O., Ali, K.A., Ezz-Elden, A., Ibrahim, M.R., Makhlouf, B.S., Hamdy, A., El Baghdady, N.S., Eldien, M.G., Allama, S., Alashkar, A.A., Seyam, A., Adel, N.A., Ibrahim, A.R.N. and Zaki, H.V. (2025), “Economic impact and clinical benefits of clinical pharmacy interventions: a six-year multi-center study using an innovative medication management tool”, PLoS One, Vol. 20 No. 1, E0311707, doi: 10.1371/journal.pone.0311707. 39823444 PMC11741631

[ref003] Anker, S.D., Butler, J., Filippatos, G., Khan, M.S., Marx, N., Lam, C.S.P., Schnaidt, S., Ofstad, A.P., Brueckmann, M., Jamal, W., Bocchi, E.A., Ponikowski, P., Perrone, S.V., Januzzi, J.L., Verma, S., Bohm, M., Ferreira, J.P., Pocock, S.J., Zannad, F. and Packer, M. (2021), “Effect of empagliflozin on cardiovascular and renal outcomes in patients with heart failure by baseline diabetes status: results from the emperor-reduced trial”, Circulation, Vol. 143 No. 4, pp. 337-349, doi: 10.1161/circulationaha.120.051824. 33175585 PMC7834911

[ref004] Behlouli, H., Feldman, D.E., Ducharme, A., Frenette, M., Giannetti, N., Grondin, F., Michel, C., Sheppard, R. and Pilote, L. (2009), “Identifying relative cut-off scores with neural networks for interpretation of the Minnesota living with heart failure questionnaire”, Annu Int Conf Ieee Eng Med Biol Soc, pp. 6242-6246. 10.1109/IEMBS.2009.5334659 19965089

[ref005] Bilbao, A., Escobar, A., Garcia-Perez, L., Navarro, G. and Quiros, R. (2016), “The Minnesota living with heart failure questionnaire: comparison of different factor structures”, Health and Quality of Life Outcomes, Vol. 14 No. 1, p. 23, doi: 10.1186/s12955-016-0425-7. 26887590 PMC4756518

[ref006] Braunschweig, F., Cowie, M.R. and Auricchio, A. (2011), “What are the costs of heart failure?”, Europace, Vol. 13 No. Suppl 2, pp. Ii13-Ii17, doi: 10.1093/europace/eur081. 21518742

[ref007] Cavallari, I., Crispino, S.P., Segreti, A., Ussia, G.P. and Grigioni, F. (2023), “Practical guidance for the use of Sglt2 inhibitors in heart failure”, American Journal of Cardiovascular Drugs, Vol. 23 No. 6, pp. 609-621, doi: 10.1007/s40256-023-00601-9. 37620653

[ref008] Docherty, K.F., Jhund, P.S., Inzucchi, S.E., Kober, L., Kosiborod, M.N., Martinez, F.A., Ponikowski, P., Demets, D.L., Sabatine, M.S., Bengtsson, O., Sjostrand, M., Langkilde, A.M., Desai, A.S., Diez, M., Howlett, J.G., Katova, T., Ljungman, C.E.A., O'meara, E., Petrie, M.C., Schou, M., Verma, S., Vinh, P.N., Solomon, S.D. and Mcmurray, J.J.V. (2020), “Effects of dapagliflozin in dapa-Hf according to background heart failure therapy”, European Heart Journal, Vol. 41 No. 25, pp. 2379-2392, doi: 10.1093/eurheartj/ehaa183. 32221582 PMC7327533

[ref009] Dokainish, H., Teo, K., Zhu, J., Roy, A., Alhabib, K.F., Elsayed, A., Palileo-Villaneuva, L., Lopez-Jaramillo, P., Karaye, K., Yusoff, K., Orlandini, A., Sliwa, K., Mondo, C., Lanas, F., Prabhakaran, D., Badr, A., Elmaghawry, M., Damasceno, A., Tibazarwa, K., Belley-Cote, E., Balasubramanian, K., Yacoub, M.H., Huffman, M.D., Harkness, K., Grinvalds, A., Mckelvie, R., Yusuf, S. and Investigators, I.-C. (2016), “Heart failure in Africa, Asia, the Middle East and South America: the inter-Chf study”, International Journal of Cardiology, Vol. 204, pp. 133-141, doi: 10.1016/j.ijcard.2015.11.183. 26657608

[ref010] El Hadidi, S. and Rosano, G. (2020), “Evidence beyond the digital medication pill”, Eur Heart J Cardiovasc Pharmacother, Vol. 6 No. 2, pp. 72-74, doi: 10.1093/ehjcvp/pvz055. 31621874

[ref011] El Hadidi, S., Darweesh, E., Byrne, S. and Bermingham, M. (2018), “A tool for assessment of heart failure prescribing quality: a systematic review and meta-analysis”, Pharmacoepidemiology and Drug Safety, Vol. 27 No. 7, pp. 685-694, doi: 10.1002/pds.4430. 29659109

[ref012] El Hadidi, S., Samir Bazan, N., Byrne, S., Darweesh, E. and Bermingham, M. (2020), “Heart failure prescribing quality at discharge from A critical care unit in Egypt: the impact of multidisciplinary care”, Pharmacy (Basel), Vol. 8 No. 3, p. 159, doi: 10.3390/pharmacy8030159. 32882858 PMC7558601

[ref013] El Hadidi, S., Vaughan, C., Kerins, D., Byrne, S., Darweesh, E. and Bermingham, M. (2021), “Guideline-led prescribing to ambulatory heart failure patients in A cardiology outpatient service”, International Journal of Clinical Pharmacy, Vol. 43 No. 4, pp. 1082-1089, doi: 10.1007/s11096-020-01220-z. 33411177

[ref014] El Hadidi, S., Hamdi, M. and Sabry, N. (2022a), “Should pharmacists lead medication reconciliation in critical care? A one-stem interventional study in an Egyptian intensive care unit”, Journal of Patient Safety, Vol. 18 No. 5, pp. E895-E899, doi: 10.1097/pts.0000000000000983. 35190512

[ref015] El Hadidi, S., Rosano, G., Tamargo, J., Agewall, S., Drexel, H., Kaski, J.C., Niessner, A., Lewis, B.S., Coats, A.J.S. and Savarese, G. (2022b), “Potentially inappropriate prescriptions in heart failure with reduced ejection fraction: Esc position statement on heart failure with reduced ejection fraction-specific inappropriate prescribing”, European Heart Journal - Cardiovascular Pharmacotherapy, Vol. 8 No. 2, pp. 187-210, doi: 10.1093/ehjcvp/pvaa108. 32941594 PMC13453067

[ref016] El-Hadidi, S., Lindberg, F., Benson, L., Uijl, A., Stolfo, D., Mol, P.G.M., Scorza, R., Cabrera, C.C., Abdin, A., Rosano, G.M.C. and Savarese, G. (2026), “Magnitude of and outcome associated with inappropriate prescribing in heart failure with reduced ejection fraction: an analysis of 50 348 patients from the Swedish heart failure registry”, European Journal of Heart Failure, Xuaf026, doi: 10.1093/ejhf/xuaf026. 41771119

[ref017] Hadidi, S.E., Bazan, N.S., Byrne, S., Darweesh, E. and Bermingham, M. (2022), “Factors influencing prescribing by critical care physicians to heart failure patients in Egypt: a cross-sectional survey”, Future Journal of Pharmaceutical Sciences, Vol. 8 No. 1, p. 40, doi: 10.1186/s43094-022-00429-1.

[ref018] Halatchev, I.G., Mcdonald, J.R. and Wu, W.C. (2020), “A patient-centred, comprehensive model for the care for heart failure: the 360 degrees heart failure centre”, Open Heart, Vol. 7 No. 2, E001221, doi: 10.1136/openhrt-2019-001221. 32624480 PMC7337888

[ref019] Hassanin, A., Hassanein, M., Bendary, A. and Maksoud, M.A. (2020), “Demographics, clinical characteristics, and outcomes among hospitalized heart failure patients across different regions of Egypt”, Egypt Heart J, Vol. 72 No. 1, p. 49, doi: 10.1186/s43044-020-00082-0. 32789717 PMC7426340

[ref020] Kosiborod, M.N., Jhund, P.S., Docherty, K.F., Diez, M., Petrie, M.C., Verma, S., Nicolau, J.C., Merkely, B., Kitakaze, M., Demets, D.L., Inzucchi, S.E., Kober, L., Martinez, F.A., Ponikowski, P., Sabatine, M.S., Solomon, S.D., Bengtsson, O., Lindholm, D., Niklasson, A., Sjostrand, M., Langkilde, A.M. and Mcmurray, J.J.V. (2020), “Effects of dapagliflozin on symptoms, function, and quality of life in patients with heart failure and reduced ejection fraction: results from the Dapa-Hf trial”, Circulation, Vol. 141 No. 2, pp. 90-99, doi: 10.1161/circulationaha.119.044138. 31736335 PMC6964869

[ref021] Lam, C.S., Teng, T.K., Tay, W.T., Anand, I., Zhang, S., Shimizu, W., Narasimhan, C., Park, S.W., Yu, C.M., Ngarmukos, T., Omar, R., Reyes, E.B., Siswanto, B.B., Hung, C.L., Ling, L.H., Yap, J., Macdonald, M. and Richards, A.M. (2016), “Regional and ethnic differences among patients with heart failure in Asia: the Asian sudden cardiac death in heart failure registry”, European Heart Journal, Vol. 37 No. 41, pp. 3141-3153, doi: 10.1093/eurheartj/ehw331. 27502121

[ref022] Mcdonagh, T.A., Metra, M., Adamo, M., Gardner, R.S., Baumbach, A., Bohm, M., Burri, H., Butler, J., Celutkiene, J., Chioncel, O., Cleland, J.G.F., Coats, A.J.S., Crespo-Leiro, M.G., Farmakis, D., Gilard, M., Heymans, S., Hoes, A.W., Jaarsma, T., Jankowska, E.A., Lainscak, M., Lam, C.S.P., Lyon, A.R., Mcmurray, J.J.V., Mebazaa, A., Mindham, R., Muneretto, C., Francesco Piepoli, M., Price, S., Rosano, G.M.C., Ruschitzka, F., Kathrine Skibelund, A., Group, E.S.C.S.D., Christian Schulze, P., Abdelhamid, M., Aboyans, V., Adamopoulos, S., Anker, S.D., Arbelo, E., Asteggiano, R., Bauersachs, J., Bayes-Genis, A., Borger, M.A., Budts, W., Cikes, M., Damman, K., Delgado, V., Dendale, P., Dilaveris, P., Drexel, H., Ezekowitz, J., Falk, V., Fauchier, L., Filippatos, G., Fraser, A., Frey, N., Gale, C.P., Gustafsson, F., Harris, J., Iung, B., Janssens, S., Jessup, M., Konradi, A., Kotecha, D., Lambrinou, E., Lancellotti, P., Landmesser, U., Leclercq, C., Lewis, B.S., Leyva, F., Linhart, A., Løchen, M.L., Lund, L.H., Mancini, D., Masip, J., Milicic, D., Mueller, C., Nef, H., Nielsen, J.C., Neubeck, L., Noutsias, M., Petersen, S.E., Sonia Petronio, A., Ponikowski, P., Prescott, E., Rakisheva, A., Richter, D.J., Schlyakhto, E., Seferovic, P., Senni, M., Sitges, M., Sousa-Uva, M., Tocchetti, C.G., Touyz, R.M., Tschoepe, C., Waltenberger, J., Baumbach, A., Chioncel, O., Coats, A.J.S., Farmakis, D., Heymans, S., Jaarsma, T., Lainscak, M., Lyon, A.R., Mebazaa, A., Muneretto, C., Price, S. and Ruschitzka, F. (2021), “2021 Esc guidelines for the diagnosis and treatment of acute and chronic heart failure”, European Heart Journal, Vol. 42 No. 36, pp. 3599-3726, doi: 10.1093/eurheartj/ehab368. 34447992

[ref023] Mcewan, P., Qin, L., Jhund, P.S., Docherty, K.F. and Mcmurray, J.J.V. (2021), “Assessing the impact of cardiovascular events on health-related quality of life outcomes in Dapa-Hf”, European Heart Journal, Vol. 42, Supplement_1, p. 907, doi: 10.1093/eurheartj/ehab724.0907.33428707

[ref024] Mcmurray, J.J., Packer, M., Desai, A.S., Gong, J., Lefkowitz, M.P., Rizkala, A.R., Rouleau, J.L., Shi, V.C., Solomon, S.D., Swedberg, K., Zile, M.R. and Investigators, P.-H. and Committees (2014), “Angiotensin-neprilysin inhibition versus enalapril in heart failure”, New England Journal of Medicine, Vol. 371 No. 11, pp. 993-1004, doi: 10.1056/nejmoa1409077. 25176015

[ref025] Monzo, L., Savarese, G., Mullens, W., Abdin, A., Bozkurt, B., Chioncel, O., El Hadidi, S., Gorter, T.M., Inciardi, R.M., Petrie, M.C., Schiattarella, G.G., Stolfo, D., Metra, M. and Girerd, N. (2025), “Pharmacological treatment for patients with obesity and heart failure: focus on glucagon-like peptide-1 receptor agonists. European journal of heart failure expert consensus document”, European Journal of Heart Failure, Vol. 27 No. 11, pp. 2465-2479, doi: 10.1002/ejhf.70082. 41309245 PMC12765371

[ref026] Moradi, M., Daneshi, F., Behzadmehr, R., Rafiemanesh, H., Bouya, S. and Raeisi, M. (2020), “Quality of life of chronic heart failure patients: a systematic review and meta-analysis”, Heart Failure Reviews, Vol. 25 No. 6, pp. 993-1006, doi: 10.1007/s10741-019-09890-2. 31745839

[ref027] Naveiro-Rilo, J.C., Diez-Juarez, D.M., Romero Blanco, A., Rebollo-Gutierrez, F., Rodriguez-Martinez, A. and Rodriguez-Garcia, M.A. (2010), “Validation of the Minnesota living with heart failure questionnaire in primary care”, Revista Espanola de Cardiologia, Vol. 63 No. 12, pp. 1419-1427, doi: 10.1016/s1885-5857(10)70276-0. 21144402

[ref028] Nicola, M., Nicola, M., Zarif, B., Ghalid, A.E., Abdelrahim, M.E.A. and Hadidi, S.E. (2024), “Prevalence of anxiety and depression and the influence of correlates in acute coronary syndrome patients: a cross-sectional analysis”, Future Journal of Pharmaceutical Sciences, Vol. 10 No. 1, p. 155, doi: 10.1186/s43094-024-00738-7.

[ref029] Oswald, A.S., Hussain, M.S., Win, M.L., Liew, Y.J., Dihoun, A., Nicholls, J., Newey, R., Baird, E., Smith, G., Garland, C., Pigazzani, F., Khan, F., Choy, A.M., Mordi, I.R. and Lang, C.C. (2024), “Blood urea monitoring in heart failure patients - an important predictor of mortality”, European Heart Journal, Vol. 45 No. 988, Ehae666, doi: 10.1093/eurheartj/ehae666.988.

[ref030] Pratley, R., Guan, X., Moro, R.J. and Do Lago, R. (2024), “Chapter 1: the burden of heart failure”, Americas Journal of Medicine, Vol. 137 No. 2, pp. S3-S8, doi: 10.1016/j.amjmed.2023.04.018. 38184324

[ref031] Severino, P., D'amato, A., Prosperi, S., Mariani, M.V., Myftari, V., Labbro Francia, A., Cestie, C., Tomarelli, E., Manzi, G., Birtolo, L.I., Marek-Iannucci, S., Maestrini, V., Mancone, M., Badagliacca, R., Fedele, F. and Vizza, C.D. (2024), “Strategy for an early simultaneous introduction of four-pillars of heart failure therapy: results from A single center experience”, American Journal of Cardiovascular Drugs, Vol. 24 No. 5, pp. 663-671, doi: 10.1007/s40256-024-00660-6. 38909334 PMC11344711

[ref032] Shchendrygina, A. and Saldarriaga, C. (2024), “New Era in heart failure management: implementing cutting-edge therapies effectively”, Open Heart, Vol. 11 No. 1, E002659, doi: 10.1136/openhrt-2024-002659. 38589204 PMC11015219

[ref033] Stolfo, D., Iacoviello, M., Chioncel, O., Anker, M.S., Bayes-Genis, A., Braunschweig, F., Cannata, A., El Hadidi, S., Filippatos, G., Jhund, P., Mebazaa, A., Moura, B., Piepoli, M., Ray, R., Ristic, A.D., Seferovic, P., Simpson, M., Skouri, H., Tocchetti, C.G., Van Linthout, S., Vitale, C., Volterrani, M., Keramida, K., Wassmann, S., Lewis, B.S., Metra, M., Rosano, G.M.C. and Savarese, G. (2025), “How to handle polypharmacy in heart failure. A clinical consensus statement of the heart failure association of the esc”, European Journal of Heart Failure, Vol. 27 No. 5, pp. 747-759, doi: 10.1002/ejhf.3642. 40091554 PMC12103960

[ref034] Tadic, S., Ilic, A., Stojsic-Milosavljevic, A., Stefanovic, M., Miljkovic, T., Bjelobrk, M., Kovacevic, M., Milovancev, A., Petrovic, M., Popov, T. and Stojsic, S. (2024), “Quality of life in patients with heart failure and improved ejection fraction: one-year follow-up with Arni and Sglt2i”, European Heart Journal, Vol. 45 No. 1043, Ehae666, doi: 10.1093/eurheartj/ehae666.1043.

[ref035] Trueman, D., Kapetanakis, V., Briggs, A., Lewis, E., Rouleau, J., Solomon, S.D., Swedberg, K., Zile, M.R., Packer, M., Mcmurray, J.J.V., Croft, D.C., Haroun, R. and Gielen, V. (2017), “P3373better health-related quality of life in patients treated with sacubitril/valsartan compared with enalapril, irrespective of Nyha class: analysis of Eq-5d in paradigm-Hf”, European Heart Journal, Vol. 38, suppl_1, Ehx504, P3373, doi: 10.1093/eurheartj/ehx504.p3373.

[ref036] Von Elm, E., Altman, D.G., Egger, M., Pocock, S.J., Gotzsche, P.C., Vandenbroucke, J.P. and Initiative, S. (2007), “Strengthening the reporting of observational studies in Epidemiology (strobe) statement: guidelines for reporting observational studies”, BMJ, Vol. 335 No. 7624, pp. 806-808, doi: 10.1136/bmj.39335.541782.ad. 17947786 PMC2034723

[ref037] Yancy, C.W., Jessup, M., Bozkurt, B., Butler, J., Casey, D.E., Jr, Colvin, M.M., Drazner, M.H., Filippatos, G.S., Fonarow, G.C., Givertz, M.M., Hollenberg, S.M., Lindenfeld, J., Masoudi, F.A., Mcbride, P.E., Peterson, P.N., Stevenson, L.W. and Westlake, C. (2017), “2017 Acc/Aha/Hfsa focused update of the 2013 Accf/Aha guideline for the management of heart failure: a report of the American College of cardiology/American heart association task force on clinical practice guidelines and the heart failure society of America”, Journal of the American College of Cardiology, Vol. 70 No. 6, pp. 776-803, doi: 10.1016/j.jacc.2017.04.025. 28461007

[ref038] Zhou, Y., Zhao, Q., Liu, Z. and Gao, W. (2024), “Blood urea nitrogen/creatinine ratio in heart failure: systematic review and meta-analysis”, PLoS One, Vol. 19 No. 5, E0303870, doi: 10.1371/journal.pone.0303870. 38805436 PMC11132513

